# Robust Automated Tumour Segmentation Network Using 3D Direction-Wise Convolution and Transformer

**DOI:** 10.1007/s10278-024-01131-9

**Published:** 2024-05-09

**Authors:** Ziping Chu, Sonit Singh, Arcot Sowmya

**Affiliations:** School of Computer Science and Engineering, UNSW Sydney, High St., Kensington, 2052 New South Wales Australia

**Keywords:** Medical image segmentation, Tumour segmentation, Convolutional Neural Networks, Vision Transformer, 3D Direction-Wise Convolution

## Abstract

Semantic segmentation of tumours plays a crucial role in fundamental medical image analysis and has a significant impact on cancer diagnosis and treatment planning. UNet and its variants have achieved state-of-the-art results on various 2D and 3D medical image segmentation tasks involving different imaging modalities. Recently, researchers have tried to merge the multi-head self-attention mechanism, as introduced by the Transformer, into U-shaped network structures to enhance the segmentation performance. However, both suffer from limitations that make networks under-perform on voxel-level classification tasks, the Transformer is unable to encode positional information and translation equivariance, while the Convolutional Neural Network lacks global features and dynamic attention. In this work, a new architecture named TCTNet *T*umour Segmentation with 3D Direction-Wise *C*onvolution and *T*ransformer) is introduced, which comprises an encoder utilising a hybrid Transformer-Convolutional Neural Network (CNN) structure and a decoder that incorporates 3D Direction-Wise Convolution. Experimental results show that the proposed hybrid Transformer-CNN network structure obtains better performance than other 3D segmentation networks on the Brain Tumour Segmentation 2021 (BraTS21) dataset. Two more tumour datasets from Medical Segmentation Decathlon are also utilised to test the generalisation ability of the proposed network architecture. In addition, an ablation study was conducted to verify the effectiveness of the designed decoder for the tumour segmentation tasks. The proposed method maintains a competitive segmentation performance while reducing computational effort by 10% in terms of floating-point operations.

## Introduction

Tumour segmentation in medical imaging is an important clinical challenge with significant implications for patient care and treatment planning [1]. Accurate segmentation of tumours from different imaging modalities, such as MRI or CT scans, is critical for accurate diagnosis, monitoring of disease progression and response to radiation therapy. The complexity of tumour morphology increases the difficulty of the segmentation process [2]. This task is further complicated by the fact that the appearance of tumours changes over time in different patients or even in the same patient [3]. Therefore, advances in tumour segmentation technology not only have the potential to improve the accuracy of diagnosis and treatment efficacy, but also play a crucial role in personalised medicine by enabling the development of targeted treatment strategies based on the different needs of patients.

With the assistance of Artificial Intelligence (AI) technologies, we can overcome the limitations of the time-consuming and labour-intensive processes associated with the task of tumour segmentation [4]. Fully Convolutional Neural Networks (FCNN) and in particular the encoder-decoder structure of UNet provide a standard method for manual medical image segmentation tasks [5, 6]. A typical ’U-shaped’ segmentation network is composed of three parts: encoder, decoder and skip connections. The encoder is responsible for extracting contextual information at different feature scales using a pyramid structure. The decoder upsamples the extracted feature representations to the required scale to obtain the voxel-level predictions. In addition, the skip connections merge the contextual information extracted by the encoder into the decoder, in order to avoid information loss during downsampling. Despite powerful representation learning capabilities, the FCNN-based methods lack long-range dependencies in the shallow layers [7]. Consequently, this leads to sub-optimal segmentation results and limited generalisation ability. Some attempts involve utilising larger convolutional kernels and atrous convolution operations to expand the receptive fields of models [8, 9]. However, the practice of enlarging the convolutional kernel size to obtain global information requires substantial computational resource requirements [10]. Recently, combinations of self-attention mechanisms and convolutional layers have been proposed to improve the global capabilities of models on image classification tasks [11, 12].

The self-attention mechanism, which is the core of the Transformer architecture, was originally designed to capture long-range linguistic information for Natural Language Processing tasks [13]. It has achieved state-of-the-art results on various sequence-to-sequence prediction tasks due to its dynamic feature extraction ability. Furthermore, using a Transformer as a backbone or encoder holds merit due to its capability of modelling long-range dependency [7]. Specifically, unlike the localised representations of convolution operations, the Vision Transformer divides a 2D/3D input image into several patches and embeds them into a 1D sequence before computing self-attention weights [14]. Such an adaptable formulation enables efficient learning of long-range information. However, the vanilla Transformer faces challenges to preserving sufficient position information due to the absence of the pyramid structure which is commonly employed in vision tasks [15]. Directly using patch embedding to transform 3D images into 1D sequences leads to poor generalisation and sub-optimal segmentation performance [16].

In this work, a hierarchical architecture is applied, while a 3D Transformer-based encoder and a 3D convolutional token embedding are utilised to obtain input sequences for a self-attention module. These modifications aim to preserve the global dynamic attention of the Transformer while acquiring the desirable proprieties of CNNs such as translation invariance. A novel decoder is designed for the Transformer-based encoder using a more efficient 3D Direction-Wise Convolution [17], aiming to enhance model performance on segmenting complex and irregular tumour boundaries. A full ablation study was established to verify the effectiveness of the encoder and the decoder structure, and to demonstrate that removing positional encoding from the proposed hybrid Transformer-CNN architecture does not affect the segmentation performance. In the end, the cropping-based training method and the sliding window inference method are shown not only to reduce the GPU memory requirement, but also to alleviate the significant data imbalance in tumour segmentation tasks.

## Related Work

### FCNN-Based Segmentation Networks

FCNN-based models, including UNet [6], have achieved state-of-the-art results on several 2D medical image segmentation benchmarks [18, 19]. Due to the limitations of GPU memory, voxel-level segmentation was initially conducted using adjacent slides to predict the classes of all voxels in the dataset. The utilisation of 3DUNet, with whole medical images as network inputs, effectively paved the way for volumetric segmentation tasks [20]. With the introduction of residual connection [21] and multi-scaled structure [22], volumetric segmentation networks obtain better generalisation ability and the capability to handle multi-scale features. nnU-Net [23], an ensemble model, performs well under different hardware conditions. Although these attempts provide spatial information at different scales for the network, they suffer from a lack of global information and long-range dependencies in the shallow layers [7]. These limitations also hinder them from achieving satisfactory performance on challenging 3D segmentation tasks.

### Transformers in Segmentation Networks

The Vision Transformer has achieved state-of-the-art results on upstream vision tasks by pre-training on large-scale datasets [24]. Unlike the vanilla Transformer, recently various pyramidal Transformers have been proposed [25, 26]. Since the hierarchical Vision Transformer extracts feature maps at various scales, it demands fewer computational resources and is more suitable for downstream vision tasks, compared to the original model. TransUNet utilises 2D CNN as the backbone and a Transformer module at the bottom-neck connection to obtain better segmentation results in 2D Brain MRI [27]. Some endeavours have embedded the feature maps obtained through convolution operations into 1D sequences and used them as inputs to the Transformer module in order to improve the capability of skip connections [28, 29]. Given the substantial GPU memory demands of both the self-attention mechanism and 3D medical images, UNETR presents an approach that involves downsampling of the image by a factor of 16 before computing the self-attention matrix [7]. CKD-TransBTS [30], which uses a hybrid Transformer-CNN architecture, employs cross-task knowledge distillation to enhance the efficiency and accuracy of Brain Tumour Segmentation in MRI scans. However, even if it gradually upsamples feature maps to the original size, it is likely to lose information at the original scale in the encoder part and may result in sub-optimal segmentation performance.

The Swin Transformer proposed a window-based multi-head self-attention and a shifted window multi-head self-attention, which makes it possible to retain global information inside a computing window [26]. SwinUNet directly uses the Swin Transformer, a variant of the Transformer, and is more applicable to downstream vision tasks in 2D medical image segmentation [31]. Hatamizadeh et al. further extended the window/shifted window multi-head attention to a 3D version and optimised the cropping training method and sliding window inference method for 3D medical image segmentation tasks [32]. They achieved state-of-the-art results on several 3D medical image segmentation benchmarks, though at a large computational cost.

## Materials and Methods

### Datasets

The Brain Tumour Segmentation 2021 dataset (BraTS21) [33] was selected for building and testing the proposed network, and two additional 3D tumour datasets were employed to compare the generalisation ability. Five-fold cross-validation was used to ensure the stability, and a fixed random seed to ensure the reproducibility of the results.

#### Brain Tumour Segmentation

**Fig. 1:**
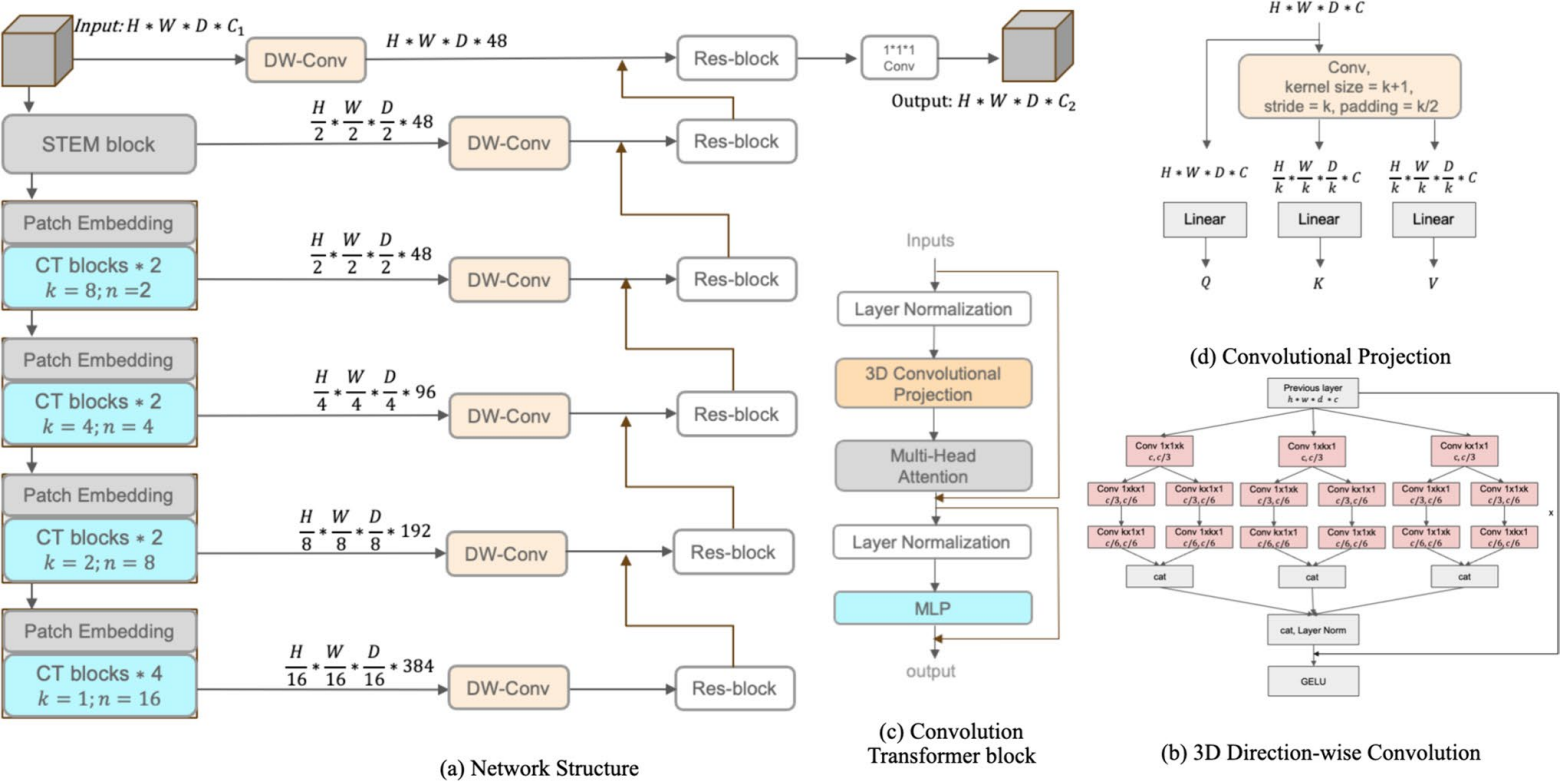
**a** Overview of TCTNet architecture. *H*, *W*, *D* stand for the size of input and output patches. $C_{1}$ and $C_{2}$ stand for the number of channels for input and output The input for Brain Tumour Segmentation to the network consists of multi-modal MRI images contains FLAIR, T1w, T1gd and T2w and the output is formed by three channels: Whole Tumour, Enhancing Tumour and Tumour Core. DW-Conv stands for 3D Direction-Wise Convolution. Inside the CT blocks, *k* stands for the reduction rate of images, and n stands for the number of heads when computing multi-head attention. **b** A more efficient implementation of 3D Direction-Wise Convolution block, GELU stands for Gaussian Error Linear Unit activation function. **c** Convolution Transformer block utilises a 3D convolutional projection to obtain Q, K, V for Multi-head Attention. **d** Convolutional Projection to obtain Q, K and V for multi-head self-attention. k means the reduction rate of images. (Best viewed in colour)

The BraTS21 challenge includes 1251 cases in the training dataset and 219 cases for testing. The original image size is $4*240*240*155$ which contains four kinds of Magnetic Resonance Imaging (MRI) modalities (FLAIR, T1w, T1gd and T2w). The MRI voxel spacing utilised for this task is $1.0*1.0*1.0 \ mm^{3}$. In this work, four channels were combined as the input and the cropping size for training and inference was $128*128*128$ as shown in Fig. 1. Initial annotations contain three sub-regions: the necrotic and non-enhancing tumour core, the peritumoral edema and the enhancing tumour. The multi-channel annotations were converted into three sub-regions based on the BraTS21 benchmark, namely Enhancing Tumour(ET), Tumour Core(TC) and Whole Tumour(WT).

#### Medical Segmentation Decathlon

Two 3D CT sub-datasets (Liver and Hepatic Vessel) from the Medical Segmentation Decathlon (MSD) [34] were employed to test the generalisation ability of the proposed network. Only the tumour annotations were retained, while the organ annotations were ignored. The Liver subset contains 121 training samples and 70 test cases. The Hepatic Vessel subset includes 303 training cases and 140 test cases. Since neither of these sub-datasets has a fixed image size, all the images were iterated over to come up with a suitable cropping size. The same cropping size was used during training and inference: $32*256*256$ for the Liver dataset and $16*256*256$ for the Hepatic Vessel dataset. This study is based on two public datasets for which no ethical approval was required.

### Methods

A segmentation network is proposed, with a hybrid Transformer-CNN encoder with the benefits of Transformer and convolutional operations, called TCTNet. Considering the complexity of tumour edges and spiculatedness [2], the decoder should be able to extract complex boundary information in every direction. The network structure is illustrated in Fig. 1 with a 3D Convolution Transformer block, a more efficient method to implement 3D Direction-Wise Convolution and convolutional projection.

#### Encoder

Since computing the multi-head self-attention requires input in the format of a 1D sequence, Transformer-based classification networks typically split an image into non-overlapping patches before embedding [35]. For Transformer-based 3D segmentation tasks, several methods employ patch embedding or linear projection directly to receive the input and reduce the computational complexity of the model [7, 32]. However, both linear projection and patch embedding are inadequate for modelling feature information within a patch, which makes the initial in-patch features insufficient in the encoder part. In order to overcome this limitation, a Stem block, which is commonly used in modern CNNs, is utilised to process the input image [36]. The Stem block consists of a $7*7*7$ convolution operation with a stride of 2 and padding of 3, along with layer normalisation and Gaussian Error Linear Unit (GELU) activation function. The introduction of the Stem block also simplifies the design, allowing the removal of positional encoding without impacting the segmentation performance. To produce the hierarchical structure, the four-stage design of modern computer vision networks is followed, and convolutional patch embedding is used for dowmsampling.

Details of the Convolution Transformer block are displayed in Fig. 1(c). The original structure of the Vision Transformer, such as the Multi-Layer Perceptron and Layer Normalisation, is not changed as the goal is to combine patients’ information into segmentation networks in a follow-up study. To mitigate GPU memory consumption, a depth-wise convolution [37] with a stride of *k* is employed to reduce resolution of feature maps used as key and value before computing multi-head self-attention. For a 1D sequence input from the previous layer $$x'\in R^{L*C}$$, where *L* stands for the length of the 1D sequence and *C* for the number of the channel. The convolution projection, detailed in Fig. 1(d), first shapes the input into a 3D image $$x\in R^{H*W*D*C}$$ where (*H*, *W*, *D*) is the size of the 3D image and satisfies $$H*W*D=L$$. After the depth-wise convolution which reduces the size of 3D images by a factor of *k*, the output image from convolution operations will be $$x^{c}\in R^{H/k * W/k * D/k * c}$$. Finally, the obtained feature map is embedded into a 1D sequence and repeated *n* times to obtain $$x^{k,v}\in R^{n, (H*W*D/k^3), C}$$, where *n* is the number of heads for computing multi-head self-attention. *n* dictates how many different representation sub-spaces that the model can focus on, and determines the complexity of the model. The query, key and value to the multi-head self-attention can be formulated as:

1 $$\begin{aligned} \left\{ \begin{aligned}x_{i}^{Q} &= x_{i-1}, \\ x_{i}^{K^{'}}&=\text {Flatten} (\text {DWConv3D} (\text {Reshape3D} (x_{i-1}))) \\ x_{i}^{V^{'}}&=\text {Flatten} (\text {DWConv3D} (\text {Reshape3D} (x_{i-1}))) \end{aligned} \right. \end{aligned}$$

Then the self-attention mechanism is applied as

2 $$\begin{aligned} \text {Attention}(Q,K,V) = \text {Softmax}(\frac{QK^{'T}}{\sqrt{d_{k}}})V^{'} \end{aligned}$$

For configuration in the encoder part, 2 blocks are applied in the first three stages and 4 blocks in the last stage. The reduction rate in each stage is 8, 4, 2 and 1 while the number of heads is 2, 4, 8, 16 separately.

#### Decoder

The decoder takes the feature maps extracted by the encoder through skip connections and fuses multi-scale features to obtain the voxel-level predictions. Existing encoders in deep Learning-based tumour segmentation networks adhere to the design for classification tasks, where the input image is directly processed by a downsampling module [4]. This design results in the omission of detailed features in the encoder part of the network [38]. The use of multiple residual blocks at various scales or maintaining feature maps at original scales is commonly used to solve this issue [32, 39]. Preserving original-size features throughout the entire process leads to excessive GPU memory usage and makes such an approach currently unsuitable for 3D segmentation tasks. Therefore, simply employing residual blocks in each layer might not adequately capture complex boundary information such as tumour edges.

Following the design of TSDNet [17], *X*, *Y*, *Z* are permuted to obtain a module with 6 sub-paths, each of which contains uni-directional convolution operations in all three axes, called 3D Direction-Wise Convolution in Fig. 1(c). A residual connection is further introduced to restrict the learning process in the 3D Direction-Wise Convolution module, which could help the module focus on the information not present in upsampled feature maps [36]. Concatenation is used to fuse the features from different sub-paths, Layer Normalisation to normalise each input in a batch independently across all features, and a GELU activation function after the identity mapping is added.

To build the decoder, 3D Direction-Wise Convolution is used in each layer after the Convolution Transformer block, and also one in the original scale, to extract complex edge features. A residual block is employed to fuse the feature maps of the current scale with the ones obtained by upsampling from the previous layer, and a $1*1*1$ Convolution with Softmax activation function is used to produce the final segmentation result.

### Implementation Details

The proposed TCTNet was implemented using Python programming language [40] and the Deep Learning frameworks Pytorch [41] and Medical Open Network for Artificial Intelligence (Monai [42]), using 4 NVIDIA V100 GPUs. Based on the $5^{th}$ and $95^{th}$ percentiles of the foreground intensities, all voxel intensities were normalised to the range [0, 1]. All models were trained for 300 epochs with a batch size of 1 in each GPU, using AdamW optimiser [43] with initial learning rate $1*10^{-4}$ and cosine annealing learning rate scheduler [44]. In addition, data augmentation techniques such as random scaling of intensity, addition of Gaussian noise, shift intensity, random rotation and random flip were applied in all three views.

A combination of random cropping and label-guided cropping was employed to obtain training patches of size $128*128*128$ from 3D input images. A sliding window inference method was applied using a Region of Interest equal to the cropping size during training. Additionally, an overlap of 0.5 and Gaussian Importance Weighting [45] were utilised to assign higher confidence to the prediction of central voxels than outside ones. For the other two tumour datasets from the Medical Segmentation Decathlon, the weight of deep layers was kept. Then fine-tuning of 200 epochs was applied to the whole network and the best model was saved based on the validation result.

To compare the consumption of computing resources, SwinUNETR is used for comparison with the proposed TCTNet. For a standard BraTS2021 input image of size $4*128*128*128$, SwinUNETR requires more than 720 Giga floating-point operations (GFlops), while TCTNet only requires 640 GFlops, which is $11\%$ less than SwinUNETR. Computational resource consumption can also be adjusted by modifying the number of dimensions of key and value as needed, to determine the number of floating-point operations required for computing the self-attention mechanism.

### Loss Function

Considering the data imbalance between tumour areas and the background, a combination of soft dice loss and cross-entropy loss was used, and it can be calculated per voxel based on formula 3:

3 $$\begin{aligned} L(G, P) = 1 - (\frac{1}{I}\sum ^{I}_{i=1}\sum ^{C}_{c=1}G_{i,c}\log P_{i,c} + \frac{2}{C}\sum ^{C}_{c=1}\frac{\sum ^{I}_{i=1}G_{i,c}P_{i,c}}{\sum ^{I}_{i=1}G^{2}_{i,c}+\sum ^{I}_{i=1}P^{2}_{i,c}}) \end{aligned}$$

where *I* denotes the number of voxels and *C* stands for the number of classes. $G_{i,c}$ and $P_{i,c}$ are the ground truth and the probability of output for voxel *i* and class *c* respectively.

### Evaluation Metrics

Following the standard metrics for semantic segmentation, the Dice Similarity Coefficient (DSC [46]) score was used to evaluate the segmentation performance of different network architectures. Let $G_{i}$ and $P_{i}$ stand for the ground truth and semantic prediction of voxel *i* respectively, then the Dice score of a 3D image can be defined in a voxel-wise manner.

## Results and Discussion

For comparison, one CNN based approach, one ensemble network and four segmentation networks that have attempted to use the multi-head self-attention were selected. The segmentation performance of all the networks for three tumour datasets are shown in Table 1. Wilcoxon signed-rank test was employed to statistically analyse the significant differences between our proposed architecture and other networks. Since there was not enough data in the original table to analyse statistically, all five-fold results were used for the Wilcoxon signed-rank test. The *p*-value obtained from the Wilcoxon signed-rank test was also recorded.

The tests between our network architecture and SegResNet, nnUNet, CoTR, TransBTS and UNETR all resulted in a *p*-value less than 0.005 ($$p < 0.005)$$. This indicates that there is a statistically significant difference between our proposed network and these method. The tests compared TCTNet with SwinUNETR and CKD-TransBTS yields a *p*-value larger than 0.005. This indicates that data did not provide sufficient evidence to conclude that our proposed method outperforms these two network architecture. The under-performance of the proposed network architecture can be attributed to its limited efficacy when dealing with small datasets.

**Table 1:** Quantitative comparison of segmentation performance on three tumour datasets: Brain Tumour Segmentation from BraTS 2021 challenge and Liver and Hepatic Vessel (HV) datasets, which are sub-datasets from the Medical Segmentation Decathlon. WT, TC and ET denote whole tumour, tumour core and enhanced tumour respectively. The best results in each column have been bolded. *p*-value is obtained by Wilcoxon signed rank test using all five-fold results

Methods	Brain Tumour (MRI)				Liver	HV	
	WT	TC	ET	Avg			*p*
SegResNet [47]	89.63	87.78	79.40	85.60	73.48	69.79	$<0.001$
nnUNet [23]	91.44	90.08	88.37	89.96	**75**.**42**	71.37	$<0.001$
CoTR [29]	90.72	88.82	82.72	87.42	71.06	71.04	$<0.001$
TransBTS [28]	90.32	89.32	83.41	87.92	72.02	70.89	$<0.001$
CKD-TransBTS [30]	92.74	**91**.**17**	87.90	90.60	74.76	72.09	$<0.01$
UNETR [7]	91.54	89.79	83.90	88.41	72.49	70.81	$<0.005$
SwinUNETR [32]	92.31	90.79	87.62	90.24	74.72	**72**.**14**	$<0.01$
TCTNet	**93**.**04**	**91**.**17**	**88**.**67**	**90**.**96**	74.81	72.07	

**Fig. 2:**
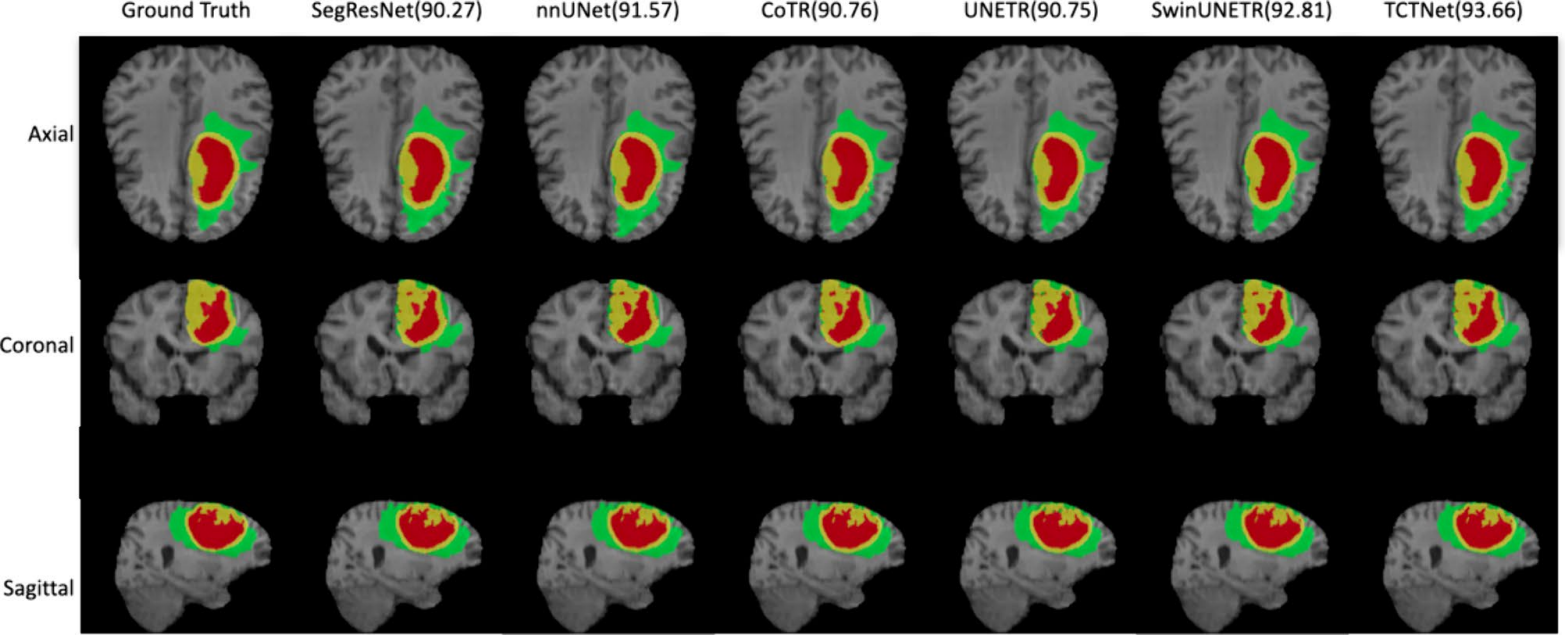
Segmentation results of networks for a specific case are presented through axial, coronal and sagittal slice. The segmentation performance in terms of Dice scores is shown at the top of each column along with the corresponding network name. (Best viewed in colour)

**Fig. 3:**
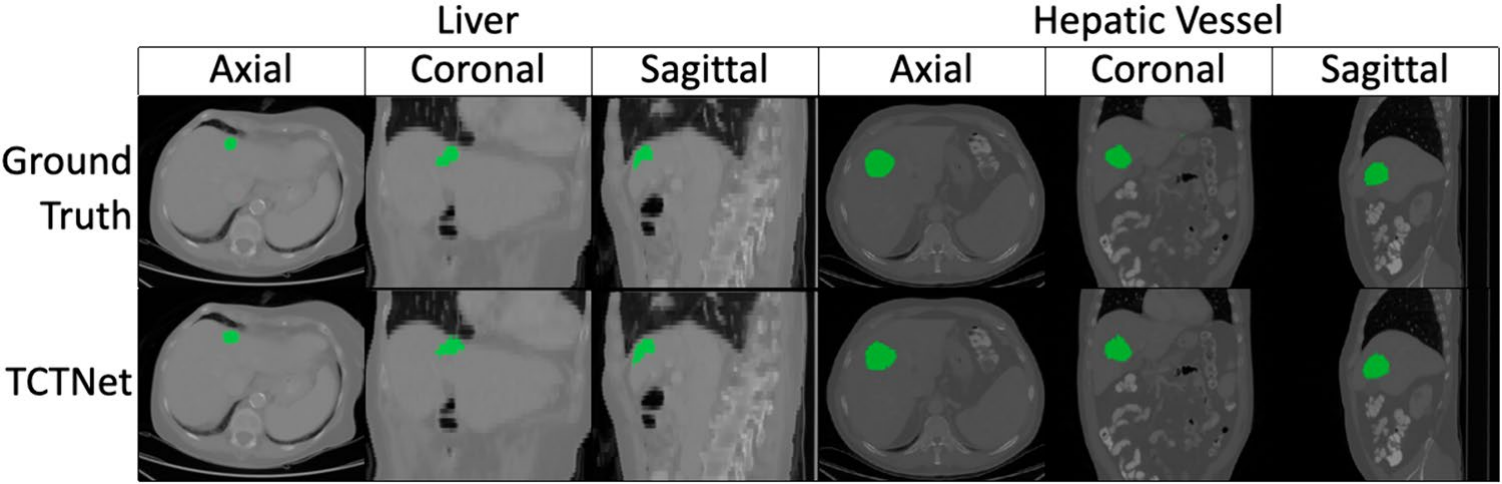
TCTNet visual segmentation results of the Liver and Hepatic Vessel datasets are shown through axial, coronal and sagittal slices. (Best viewed in colour)

Due to the limited amount of data in the liver and hepatic vessel datasets, the visualisation segmentation results are not satisfactory. It is difficult to qualitatively compare the differences in segmentation results for different network architectures. Therefore, the Brain Tumour Segmentation 2021 is used for qualitative comparison. Visualisation of segmentation results using TCTNet and other network architectures for the same case are demonstrated in Fig. 2 along with the ground truth. In addition, the ground truth of some cases in the Liver and Hepatic Vessel datasets as well as the predictive segmentation results of our network are presented in Fig. 3. For all the segmentation results, the axial, coronal and sagittal sections passing through the same point were selected. The segmentation performance of the networks for this particular case, in terms of the Dice score, is displayed at the top of the columns.

### Training and Inference Method

In the initial attempts, the entire multi-modality input was resized into an affordable size for the network and the output scaled to the desired shape. However, this approach did not perform well. Therefore, experiments with the proposed network structure were conducted to analyse various methods of training and inference on the Brain tumour 2021 dataset. Cropping-base training with sliding window inference was used as the baseline, and overlapping and Gaussian importance weight were added to the inference part step-by-step. The results of resizing twice were also recorded for comparison. All results were obtained using five-fold cross-validation and recorded in Table 2.

**Table 2:** Different training and inference methods. s.w. stands for sliding window inference, o.l. denotes overlapping of 0.5. g.m. stands for adding a Gaussian Importance map. WT, TC and ET denote whole tumour, tumour core and enhanced tumour respectively. *p*-value is obtained by Wilcoxon signed-rank test using all five-fold results

Methods	WT	TC	ET	Average
Resize twice	80.32	76.24	74.70	77.08
Crop+s.w.	88.35	84.32	80.39	84.35
Crop+s.w.+o.l.	90.34	88.91	84.61	87.95
Crop+s.w.+o.l.+g.m.	**93.04**	**91.17**	**88.67**	**90.96**

Bold emphasise best results in terms of Dice Similarity Coefficient (DSC)

The results show that the sliding window inference method was able to classify tumour voxels more accurately with the assistance of overlapping and Gaussian importance maps. The sliding window inference method was initially proposed to mitigate the problem of 3D images that cannot be loaded into the network directly due to GPU memory limitations [48]. The integration of overlapping and Gaussian importance map alleviates the issue of insufficient neighbourhood information to classify the edge voxels within windows.

**Fig. 4:**
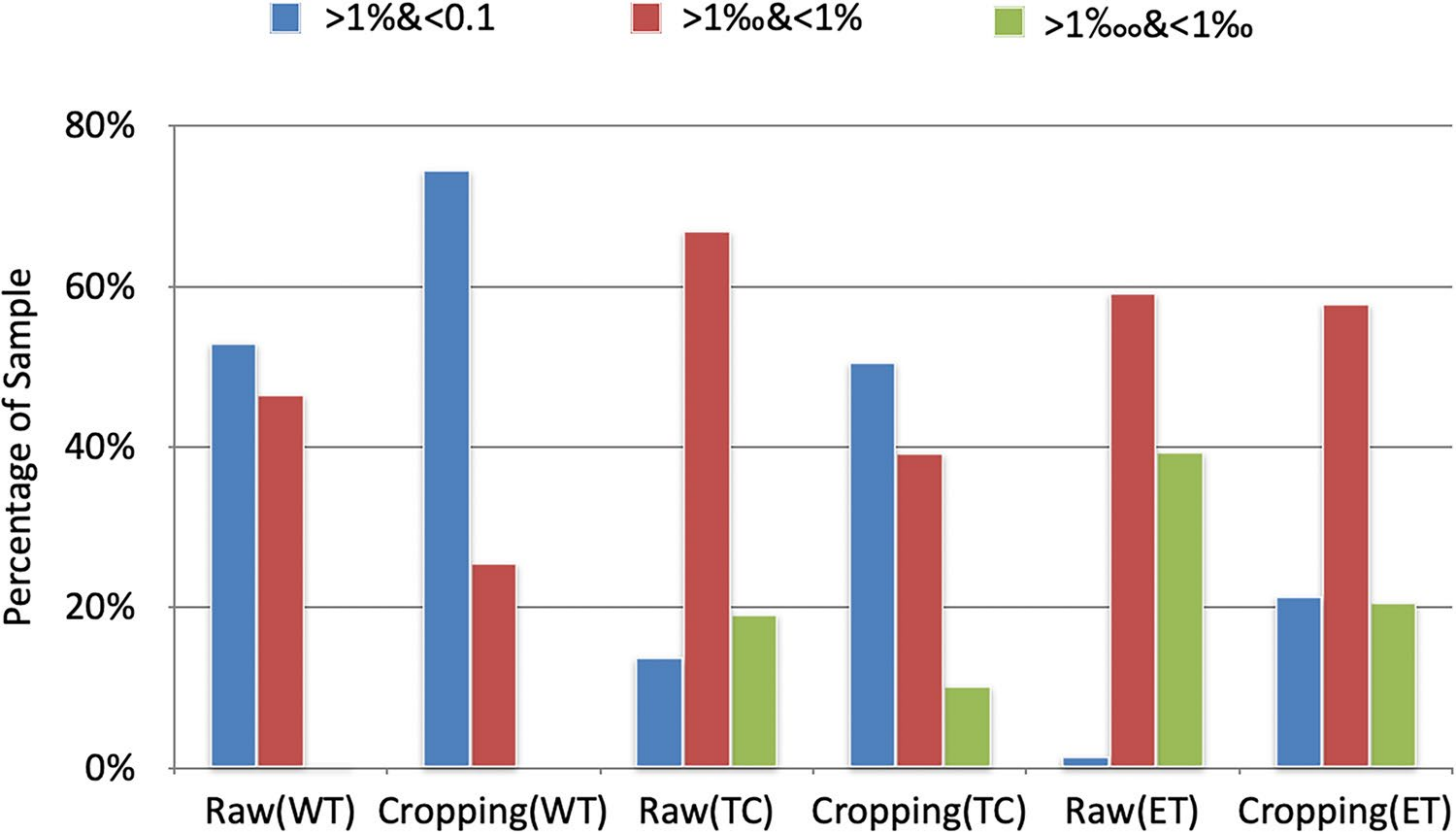
Ratio of tumours in 3D images. Raw represents the original MRI images. Cropping stands for training images obtained using a combination of random cropping and around-label cropping. WT, TC and ET denote the three brain tumour sub-regions, namely Whole Tumour, Tumour Core and Enhancing Tumour. (Best viewed in colour)

The patch-based training approach also mitigates the class imbalance between the tumour region and the background. The ratios of the tumour in the original 3D MRI images from the Brain Tumour Segmentation dataset and the training patches obtained using a combination of random cropping and around-label cropping are shown in a bar graph in Fig. 4. All images were cropped 20 times (10 times of random cropping and 10 times of cropping around-label area) to avoid the data obtained from cropping being accidental. The results indicate that this approach alleviates the class imbalance between tumour regions and the backgrounds, while also ensuring that the network has seen all parts of the 3D brain MRI scans.

### Ablation Study

Ablation studies were conducted on the proposed decoder and to address the necessity of positional coding.

#### Encoder

A Standard encoder built by Residual blocks from SegResNet [21] is utilised to demonstrate the effectiveness of the proposed hybrid encoder structure. Five-fold cross-validation was applied and the segmentation performance of Brain Tumour Segmentation task is recorded in Table 3. A Wilcoxon signed-rank test was conducted to assess the statistical significance of the segmentation performance of the proposed encoder, based on results from five different tests. The test yielded a *p*-value less than 0.001 which indicates that the results are statistically significant. Therefore, the proposed hybrid Transformer-CNN encoder performs better than standard encoder structure in segmenting brain tumours.

**Table 3:** Ablation study on the encoder part using BraTS dataset. Res. stands for the network architecture using residual blocks as the encoder. Segmentation performance was recorded using the Dice Similarity Coefficient. WT, TC and ET denote whole tumour, tumour core and enhanced tumour respectively. *p*-value is obtained by Wilcoxon signed-rank test using all five-fold results

Methods	WT	TC	ET	Average	*p*-value
TCTNet w. Res.	87.72	87.07	85.47	86.75	$<0.005$
TCTNet	**93.04**	**91.17**	**88.67**	**90.96**	

Bold emphasise best results in terms of Dice Similarity Coefficient (DSC)

#### Decoder

Residual blocks were employed to replace the 3D Direction-Wise Convolution block in the proposed TCTNet and trained on BraTS dataset. Five-fold cross-validation was applied and the segmentation results are recorded in Table 4. Wilcoxon signed-rank test was employed to evaluate the statistical significance of segmentation performance of the proposed encoder, based on all five-fold results. The obtained *p*-value is less than 0.005 which suggests significant statistical results. This indicates that the proposed 3D Direction-Wise Convolution could assist in segmenting complex and irregular brain tumour boundaries.

**Table 4:** Ablation study on decoder which utilises 3D Direction-Wise Convolution, using BraTS dataset. Segmentation performance was recorded using the Dice score. DWC stands for 3D Direction-Wise Convolution. WT, TC and ET denote whole tumour, tumour core and enhanced tumour respectively. *p*-value is obtained by Wilcoxon signed-rank test using all five-fold results

Methods	WT	TC	ET	Average	*p*-value
TCTNet w.o. DWC	91.72	90.71	87.63	90.02	$<0.005$
TCTNet	**93.04**	**91.17**	**88.67**	**90.96**	

Bold emphasise best results in terms of Dice Similarity Coefficient (DSC)

#### Positional Encoding

The network design was simplified by removing the positional encoding in the original Transformer structure. Experiments were conducted to demonstrate that removing the positional encoding does not affect network performance on segmenting brain tumours. Relative positional encoding was applied and compared to absolute positional encoding, and the network was trained and tested using five-fold cross-validation. All results are recorded in Table 5. Two Wilcoxon signed-rank tests were conducted using all five-fold results to evaluate the statistical significance of the segmentation performance of model with different positional encoding methods. The obtained *p*-value are recorded in Table 5. The results suggest that eliminating relative positional encoding does not affect the performance of the model in segmenting brain tumours. In addition, the incorporation of absolute positional encoding leads to a worse segmentation performance, which is shown by the lower Dice scores.

**Table 5:** Ablation study on positional encoding, where ape. stands for absolute positional encoding and rpe. denotes relative positional encoding. WT, TC and ET denote whole tumour, tumour core and enhanced tumour respectively. *p*-value is obtained by Wilcoxon signed-rank test using all five-fold results

Methods	WT	TC	ET	Average	*p*-value
TCTNet w. ape.	91.43	89.67	85.31	88.80	$<0.001$
TCTNet w. rpe.	**93.10**	**91.19**	88.63	**90.97**	$>0.1$
TCTNet	93.04	91.17	**88.67**	90.96	

Bold emphasise best results in terms of Dice Similarity Coefficient (DSC)

The results also indicate that the use of the Stem block and convolutional projection could simplify the design of Transformer-based medical image segmentation networks by removing positional encoding.

### Discussion

In this work, an hybrid Transformer-CNN structure with 3D Direction-Wise Convolutions is proposed to segment complex and irregular tumour boundaries and achieved state-of-the-art results on segmentation brain tumours. Compared with other Transformer-based medical image segmentation networks, the proposed TCTNet could and extract more boundary information and also eliminate positional encoding without affecting the segmentation performance. Even though the proposed network performs well on large annotated datasets, it suffers from the following three limitations.

- To achieve great segmentation performance, the proposed TCTNet requires a large amount of labelled data and its performance on small datasets is weaker than other state-of-the-art network architectures.
- The computation of the self-attention mechanism still involves converting 3D medical images into 1D sequences, which could lead to a lack of spatial information.
- The proposed network architecture has not been evaluated in the context of unsupervised learning. In practical clinical settings, the availability of large labelled dataset for training segmentation networks is often limited. Therefore, few-shot learning is also a focus of our future research.

## Conclusion

Segmentation of tumours in medical imaging modalities is challenging due to the significant data imbalance between tumour and the background. In this work, a hybrid Transformer-CNN segmentation network called TCTNet has been proposed. TCTNet includes a Transformer-based encoder which follows the structure design of the vanilla Transformer block; however, it uses convolutional embedding instead of patch embedding and a Stem block to process input images. These changes allow the Transformer-based segmentation network to remove positional encoding without reducing the performance. A decoder has been introduced that utilises a more efficient 3D Direction-Wise Convolution for further enhancing the segmentation network’s ability to model complex tumour boundaries. In addition, the utilisation of cropping-based training and sliding window inference were analysed with the assistance of overlapping and Gaussian importance weights. Future work will attempt to incorporate patient information to build multi-modal networks to further improve segmentation performance and to add unsupervised learning for utilising unlabelled medical images.

## Data Availability

The data that support the findings of this study is publicly available as part of the RSNA-ANSR-MICCAI Brain Tumor Segmentation (BraTS) 2021 Challenge which can be downloaded from http://braintumorsegmentation.org.

## References

[1] Yang, R., Yu, Y.: Artificial convolutional neural network in object detection and semantic segmentation for medical imaging analysis. Frontiers in oncology **11**, 638182 (2021) 10.3389/fonc.2021.638182 PMC7986719 33768000

[2] Limkin, E.J., Reuzé, S., Carré, A., Sun, R., Schernberg, A., Alexis, A., Deutsch, E., Ferté, C., Robert, C.: The complexity of tumor shape, spiculatedness, correlates with tumor radiomic shape features. Scientific reports **9**(1), 1–12 (2019) 10.1038/s41598-019-40437-5 PMC6416263 30867443

[3] Fingeret, M.C., Teo, I., Epner, D.E.: Managing body image difficulties of adult cancer patients: lessons from available research. Cancer **120**(5), 633–641 (2014) 10.1002/cncr.28469 PMC4052456 24895287

[4] Shi, Z., Miao, C., Schoepf, U.J., Savage, R.H., Dargis, D.M., Pan, C., Chai, X., Li, X.L., Xia, S., Zhang, X., *et al*: A clinically applicable deep-learning model for detecting intracranial aneurysm in computed tomography angiography images. Nature communications **11**(1), 6090 (2020) 10.1038/s41467-020-19527-w PMC7705757 33257700

[5] Long, J., Shelhamer, E., Darrell, T.: Fully convolutional networks for semantic segmentation. In: Proceedings of the IEEE Conference on Computer Vision and Pattern Recognition, pp. 3431–3440 (2015) 10.1109/TPAMI.2016.2572683 27244717

[6] Ronneberger, O., Fischer, P., Brox, T.: U-net: Convolutional networks for biomedical image segmentation. In: Medical Image Computing and Computer-Assisted Intervention–MICCAI 2015: 18th International Conference, Munich, Germany, October 5-9, 2015, Proceedings, Part III 18, pp. 234–241 (2015). Springer

[7] Hatamizadeh, A., Tang, Y., Nath, V., Yang, D., Myronenko, A., Landman, B., Roth, H.R., Xu, D.: Unetr: Transformers for 3d medical image segmentation. In: Proceedings of the IEEE/CVF Winter Conference on Applications of Computer Vision, pp. 574–584 (2022)

[8] Chen, L.-C., Papandreou, G., Kokkinos, I., Murphy, K., Yuille, A.L.: Deeplab: Semantic image segmentation with deep convolutional nets, atrous convolution, and fully connected crfs. IEEE transactions on pattern analysis and machine intelligence **40**(4), 834–848 (2017) 10.1109/TPAMI.2017.2699184 28463186

[9] Chen, L.-C., Papandreou, G., Schroff, F., Adam, H.: Rethinking atrous convolution for semantic image segmentation. arXiv preprint arXiv:1706.05587 (2017)

[10] Chen, L.-C., Zhu, Y., Papandreou, G., Schroff, F., Adam, H.: Encoder-decoder with atrous separable convolution for semantic image segmentation. In: Proceedings of the European Conference on Computer Vision (ECCV), pp. 801–818 (2018)

[11] Zhang, H., Goodfellow, I., Metaxas, D., *et al*: Odena. self-attention generative adversarial network. In: Proc. Int. Conf. Mach. Learn, pp. 7354–7363 (2019)

[12] Wu, H., Xiao, B., Codella, N., Liu, M., Dai, X., Yuan, L., Zhang, L.: Cvt: Introducing convolutions to vision transformers. In: Proceedings of the IEEE/CVF International Conference on Computer Vision, pp. 22–31 (2021)

[13] Vaswani, A., Shazeer, N., Parmar, N., Uszkoreit, J., Jones, L., Gomez, A.N., Kaiser, Ł., Polosukhin, I.: Attention is all you need. Advances in neural information processing systems **30** (2017)

[14] Park, N., Kim, S.: How do vision transformers work? arXiv preprint arXiv:2202.06709 (2022)

[15] Khan, S., Naseer, M., Hayat, M., Zamir, S.W., Khan, F.S., Shah, M.: Transformers in vision: A survey. ACM computing surveys (CSUR) **54**(10s), 1–41 (2022)

[16] Zheng, S., Lu, J., Zhao, H., Zhu, X., Luo, Z., Wang, Y., Fu, Y., Feng, J., Xiang, T., Torr, P.H., *et al*: Rethinking semantic segmentation from a sequence-to-sequence perspective with transformers. In: Proceedings of the IEEE/CVF Conference on Computer Vision and Pattern Recognition, pp. 6881–6890 (2021)

[17] Chu, Z., Singh, S., Sowmya, A.: TSDNET: A tumour segmentation network with 3d direction-wise convolution. In: 2023 IEEE 20th International Symposium on Biomedical Imaging (ISBI), pp. 1–5 (2023). IEEE

[18] Zhou, Z., Rahman Siddiquee, M.M., Tajbakhsh, N., Liang, J.: Unet++: A nested u-net architecture for medical image segmentation. In: Deep Learning in Medical Image Analysis and Multimodal Learning for Clinical Decision Support: 4th International Workshop, DLMIA 2018, and 8th International Workshop, ML-CDS 2018, Held in Conjunction with MICCAI 2018, Granada, Spain, September 20, 2018, Proceedings 4, pp. 3–11 (2018). Springer 10.1007/978-3-030-00889-5_1 PMC7329239 32613207

[19] Xiao, X., Lian, S., Luo, Z., Li, S.: Weighted res-unet for high-quality retina vessel segmentation. In: 2018 9th International Conference on Information Technology in Medicine and Education (ITME), pp. 327–331 (2018). IEEE

[20] Çiçek, Ö., Abdulkadir, A., Lienkamp, S.S., Brox, T., Ronneberger, O.: 3d u-net: learning dense volumetric segmentation from sparse annotation. In: Medical Image Computing and Computer-Assisted Intervention–MICCAI 2016: 19th International Conference, Athens, Greece, October 17-21, 2016, Proceedings, Part II 19, pp. 424–432 (2016). Springer

[21] Yang, J., Wu, B., Li, L., Cao, P., Zaiane, O.: Msds-unet: A multi-scale deeply supervised 3d u-net for automatic segmentation of lung tumor in ct. Computerized Medical Imaging and Graphics **92**, 101957 (2021) 10.1016/j.compmedimag.2021.101957 34325225

[22] Roth, H.R., Oda, H., Hayashi, Y., Oda, M., Shimizu, N., Fujiwara, M., Misawa, K., Mori, K.: Hierarchical 3d fully convolutional networks for multi-organ segmentation. arXiv preprint arXiv:1704.06382 (2017)

[23] Isensee, F., Jaeger, P.F., Kohl, S.A., Petersen, J., Maier-Hein, K.H.: nnu-net: a self-configuring method for deep learning-based biomedical image segmentation. Nature methods **18**(2), 203–211 (2021) 10.1038/s41592-020-01008-z 33288961

[24] Dosovitskiy, A., Beyer, L., Kolesnikov, A., Weissenborn, D., Zhai, X., Unterthiner, T., Dehghani, M., Minderer, M., Heigold, G., Gelly, S., et al.: An image is worth 16x16 words: Transformers for image recognition at scale. arXiv preprint arXiv:2010.11929 (2020)

[25] Wang, W., Xie, E., Li, X., Fan, D.-P., Song, K., Liang, D., Lu, T., Luo, P., Shao, L.: Pyramid vision transformer: A versatile backbone for dense prediction without convolutions. In: Proceedings of the IEEE/CVF International Conference on Computer Vision, pp. 568–578 (2021)

[26] Liu, Z., Lin, Y., Cao, Y., Hu, H., Wei, Y., Zhang, Z., Lin, S., Guo, B.: Swin transformer: Hierarchical vision transformer using shifted windows. In: Proceedings of the IEEE/CVF International Conference on Computer Vision, pp. 10012–10022 (2021)

[27] Chen, J., Lu, Y., Yu, Q., Luo, X., Adeli, E., Wang, Y., Lu, L., Yuille, A.L., Zhou, Y.: TransUNet: Transformers make strong encoders for medical image segmentation. arXiv preprint arXiv:2102.04306 (2021)

[28] Wang, W., Chen, C., Ding, M., Yu, H., Zha, S., Li, J.: TransBTS: Multimodal brain tumor segmentation using transformer. In: Medical Image Computing and Computer Assisted Intervention–MICCAI 2021: 24th International Conference, Strasbourg, France, September 27–October 1, 2021, Proceedings, Part I 24, pp. 109–119 (2021). Springer

[29] Xie, Y., Zhang, J., Shen, C., Xia, Y.: Cotr: Efficiently bridging cnn and transformer for 3d medical image segmentation. In: Medical Image Computing and Computer Assisted Intervention–MICCAI 2021: 24th International Conference, Strasbourg, France, September 27–October 1, 2021, Proceedings, Part III 24, pp. 171–180 (2021). Springer

[30] Lin, J., Lin, J., Lu, C., Chen, H., Lin, H., Zhao, B., Shi, Z., Qiu, B., Pan, X., Xu, Z., et al.: Ckd-transbts: clinical knowledge-driven hybrid transformer with modality-correlated cross-attention for brain tumor segmentation. IEEE transactions on medical imaging (2023) 10.1109/TMI.2023.3250474 37027751

[31] Cao, H., Wang, Y., Chen, J., Jiang, D., Zhang, X., Tian, Q., Wang, M.: Swin-Unet: Unet-like pure transformer for medical image segmentation. In: European Conference on Computer Vision, pp. 205–218 (2022). Springer

[32] Hatamizadeh, A., Nath, V., Tang, Y., Yang, D., Roth, H.R., Xu, D.: Swin UNETR: Swin transformers for semantic segmentation of brain tumors in MRI images. In: International MICCAI Brainlesion Workshop, pp. 272–284 (2021). Springer

[33] Baid, U., Ghodasara, S., Mohan, S., Bilello, M., Calabrese, E., Colak, E., Farahani, K., Kalpathy-Cramer, J., Kitamura, F.C., Pati, S., et al.: The rsna-asnr-miccai brats 2021 benchmark on brain tumor segmentation and radiogenomic classification. arXiv preprint arXiv:2107.02314 (2021)

[34] Antonelli, M., Reinke, A., Bakas, S., Farahani, K., Kopp-Schneider, A., Landman, B.A., Litjens, G., Menze, B., Ronneberger, O., Summers, R.M., *et al*: The medical segmentation decathlon. Nature communications **13**(1), 4128 (2022) 10.1038/s41467-022-30695-9 PMC9287542 35840566

[35] Touvron, H., Cord, M., Douze, M., Massa, F., Sablayrolles, A., Jégou, H.: Training data-efficient image transformers & distillation through attention. In: International Conference on Machine Learning, pp. 10347–10357 (2021). PMLR

[36] He, K., Zhang, X., Ren, S., Sun, J.: Deep residual learning for image recognition. In: Proceedings of the IEEE Conference on Computer Vision and Pattern Recognition, pp. 770–778 (2016)

[37] Xie, S., Girshick, R., Dollár, P., Tu, Z., He, K.: Aggregated residual transformations for deep neural networks. In: Proceedings of the IEEE Conference on Computer Vision and Pattern Recognition, pp. 1492–1500 (2017)

[38] Badrinarayanan, V., Kendall, A., Cipolla, R.: Segnet: A deep convolutional encoder-decoder architecture for image segmentation. IEEE transactions on pattern analysis and machine intelligence **39**(12), 2481–2495 (2017) 10.1109/TPAMI.2016.2644615 28060704

[39] Wang, J., Sun, K., Cheng, T., Jiang, B., Deng, C., Zhao, Y., Liu, D., Mu, Y., Tan, M., Wang, X., *et al*: Deep high-resolution representation learning for visual recognition. IEEE transactions on pattern analysis and machine intelligence **43**(10), 3349–3364 (2020) 10.1109/TPAMI.2020.2983686 32248092

[40] Van Rossum, G., Drake, F.L.: Python 3 Reference Manual. CreateSpace, Scotts Valley, CA (2009)

[41] Paszke, A., Gross, S., Massa, F., Lerer, A., Bradbury, J., Chanan, G., Killeen, T., Lin, Z., Gimelshein, N., Antiga, L., et al.: Pytorch: An imperative style, high-performance deep learning library. Advances in neural information processing systems **32** (2019)

[42] Cardoso, M.J., Li, W., Brown, R., Ma, N., Kerfoot, E., Wang, Y., Murrey, B., Myronenko, A., Zhao, C., Yang, D., et al.: MONAI: An open-source framework for deep learning in healthcare. arXiv preprint arXiv:2211.02701 (2022)

[43] Loshchilov, I., Hutter, F.: Decoupled weight decay regularization. arXiv preprint arXiv:1711.05101 (2017)

[44] Loshchilov, I., Hutter, F.: SGDR: Stochastic gradient descent with warm restarts. arXiv preprint arXiv:1608.03983 (2016)

[45] Salimbeni, H., Dutordoir, V., Hensman, J., Deisenroth, M.: Deep gaussian processes with importance-weighted variational inference. In: International Conference on Machine Learning, pp. 5589–5598 (2019). PMLR

[46] Zou, K.H., Warfield, S.K., Bharatha, A., Tempany, C.M., Kaus, M.R., Haker, S.J., Wells III, W.M., Jolesz, F.A., Kikinis, R.: Statistical validation of image segmentation quality based on a spatial overlap index1: scientific reports. Academic radiology **11**(2), 178–189 (2004) 10.1016/S1076-6332(03)00671-8 PMC1415224 14974593

[47] Myronenko, A.: 3D MRI brain tumor segmentation using autoencoder regularization. In: International MICCAI Brainlesion Workshop, pp. 311–320 (2018). Springer

[48] Felzenszwalb, P.F., Girshick, R.B., McAllester, D.: Cascade object detection with deformable part models. In: 2010 IEEE Computer Society Conference on Computer Vision and Pattern Recognition, pp. 2241–2248 (2010). IEEE

